# Brassinosteroids: A Promising Option in Deciphering Remedial Strategies for Abiotic Stress Tolerance in Rice

**DOI:** 10.3389/fpls.2017.02151

**Published:** 2017-12-20

**Authors:** Isha Sharma, Navdeep Kaur, Pratap K. Pati

**Affiliations:** Department of Biotechnology, Guru Nanak Dev University, Amritsar, India

**Keywords:** rice, phytohormones, brassinosteroids, abiotic stress, stress amelioration, reactive oxygen species, signal transduction

## Abstract

Rice is an important staple crop as it feeds about a half of the earth’s population. It is known to be sensitive to a range of abiotic stresses which result in significant decline in crop productivity. Recently, the use of phytohormones for abiotic stress amelioration has generated considerable interest. Plants adapt to various environmental stresses by undergoing series of changes at physiological and molecular levels which are cooperatively modulated by various phytohormones. Brassinosteroids (BRs) are a class of naturally occurring steroidal phytohormones, best known for their role in plant growth and development. For the past two decades, greater emphasis on studies related to BRs biosynthesis, distribution and signaling has resulted in better understanding of BRs function. Recent advances in the use of contemporary genetic, biochemical and proteomic tools, with a vast array of accessible biological resources has led to an extensive exploration of the key regulatory components in BR signaling networks, thus making it one of the most well-studied hormonal pathways in plants. The present review highlights the advancements of knowledge in BR research and links it with its growing potential in abiotic stress management for important crop like rice.

## Introduction

Rice is an important food crop, produced in many countries worldwide and feeding significant portions of the global population (IRRI, 2006; Kaur and Pati, 2017). More than half of the world’s population acquires 80% of their calories from rice (FAO, 2008). However, its cultivation and yield is under tremendous pressure due to adverse environmental stresses. It is sensitive to a range of abiotic stresses, including salinity, drought, submersion and cold (Wani and Sah, 2014). It is estimated that worldwide around 30–60% of yield loss per annum in rice occurs due to various kind of stresses (Seo et al., 2011). To reduce the impact of these stresses in rice plants, in depth analysis of the associated responses to stresses have been given priority in the past (Kumar et al., 2013; Mizoi and Yamaguchi-Shinozaki, 2013).

For many decades, conscious efforts have been made to overcome negative effects of environmental stresses such as remedial water management, use of tolerant cultivars and divergent cultural practices (Parry et al., 2005; Neumann, 2008). Other approaches adopted involve plant breeding which create useful genetic variations to withstand a specific environmental stress (Hu and Xiong, 2014; Mickelbart et al., 2015). However, the process of breeding tolerant rice cultivars is very slow owing to numerous concerns including a paucity of knowledge of the mechanisms underlying tolerance, intricacies of the traits associated with the stress, inadequate selection criteria and absence of rigorous, consistent and reproducible screening methodologies (Gregorio et al., 2002). Moreover, attempts to improve stress tolerance through conventional plant breeding methods are time consuming, laborious and dependent on existing genetic variability. Classical genetics suggests that stress tolerance is generally a multigenic trait (Barton and Turelli, 1990; Hoffmann and Parsons, 1991) and therefore, it is difficult to obtain precise control of the traits that the host plant will express or consistent genetic stability in subsequent generations. Further, the complexity of traits involved in tolerance of a particular stress and the coexistence of various types of environmental challenges in nature, exacerbates the magnitude of the problem and significantly impedes progression in the development of tolerant varieties (Ismail et al., 2007). Progress in the field of molecular biology has resulted in identification and use of molecular markers to expedite the breeding program for conferring abiotic stress tolerance in plants (Gregorio et al., 2013; Ismail and Horie, 2017). However, it was observed that the introgression of genomic segments (Quantitative trait loci, QTLs) attributed to stress tolerance might also be responsible for bringing undesirable traits from the donor plants (Bhatnagar-Mathur et al., 2008). To overcome these problems, development of genetically engineered plants by insertion of stress responsive and putative tolerance conferring genes has attracted a lot of attention worldwide (Bhatnagar-Mathur et al., 2008; Saad et al., 2013). There are several reports of use of gene transfer technology to alter the accumulation of osmoprotectants, increase the production of chaperones, enhance the free radical scavenging and exclusion, or compartmentalization of ions employing competent transporter and symporter systems to confer abiotic stress tolerance in rice (Garg et al., 2002; Chen et al., 2008; Zhao et al., 2009; Liu A.L. et al., 2013; Todaka et al., 2015). Recently, there has been a paradigm shift from gene centric to genome centric approach in crop improvement program. In this regard, manipulation of the cellular regulatory machinery containing transcription factors are given preference over the attempts to insert “single-action” genes (Khong et al., 2008). However, evaluation of transgenic plants for stress tolerance, and prediction/detection of the physiological effect of the insertions at the whole plant level remains as a major challenge. Besides this, the transgenic approach to offer tolerance to plants faces skepticism due to various concerns such as the possible impact of transgenic plants on biodiversity, cross-contamination of germplasm with transgenic material and lack of public acceptance of transgenic food due to potential health risks. In this scenario, escalating the stress-resistance of plants through non-transgenic means could be one of the most effective measures to solve the worldwide problem (Sharma et al., 2015; Divi et al., 2016). Under these circumstances, scientists are being compelled to develop innovative approaches which could be widely accepted to public for global agriculture. In this context, the essential role of phytohormones in stress regulatory processes of plant is being explored in various laboratories across the world. Although, phytohormones are usually synthesized at low concentration in plants, however, they control plethora of developmental events in a plant under normal conditions. Phytohormones control plant’s functions by regulating their own biosynthesis, modulating their available pool for a particular action, or influencing various signaling cascades.

## BRs: A Promising Phytohormone in Abiotic Stress Amelioration

Brassinosteroids are important phytohormones due to their versatile roles in plants (Vardhini, 2016; Wei and Li, 2016; Yusuf et al., 2017). They are plant-specific steroidal hormones which are perceived by a cell-surface receptor family of leucine-rich repeat receptor kinases BRASSINOSTEROID INSENSITIVE 1 (BRI1), which interacts with co-receptor BRI1 ASSOCIATED RECEPTOR KINASE 1 (BAK1) and undergoes a series of phosphorylation and dephosphorylation events to transduce information to the nucleus that results in the regulation of expression of several hundred genes involved in diverse physiological functions (Sharma et al., 2013c; Belkhadir and Jaillais, 2015; Nakamura et al., 2017). BRs regulate different forms of plant growth and development including xylem differentiation, photomorphogenesis, cell elongation and seed germination (Vriet et al., 2012; Zhang et al., 2014). However, recently, there has been a prominent increase in their application in agricultural processes to improve crop productivity under stress and native conditions (Vriet et al., 2012; Zhang et al., 2014; Marakli and Gozukirmizi, 2016; Liu et al., 2017). Keeping in mind the scope of BRs in agriculture, in the past few years, efforts have been undertaken to gain better insights into BR metabolism and signaling (Belkhadir and Jaillais, 2015). Although several studies have been conducted on the potential of BRs for enhancing stress tolerance (Zhang et al., 2014; Ahammed et al., 2015; Sharma et al., 2015; Hegazi et al., 2017; Wang et al., 2017), yet their underlying mechanisms of action remain elusive. This could be due to the integration of BR signals in many other signaling networks linked to stress mitigation (Divi and Krishna, 2010; Ahammed et al., 2015; Divi et al., 2016). The present review discusses some of the possible mechanisms through which BRs could modulate stress responses in plants. The knowledge gained from this can be extrapolated for deciphering remedial strategies for abiotic stress tolerance in hugely important crops like rice.

## BRs Mediated Stress Responses Occur at Different Levels of Organization

**FIGURE 1:**
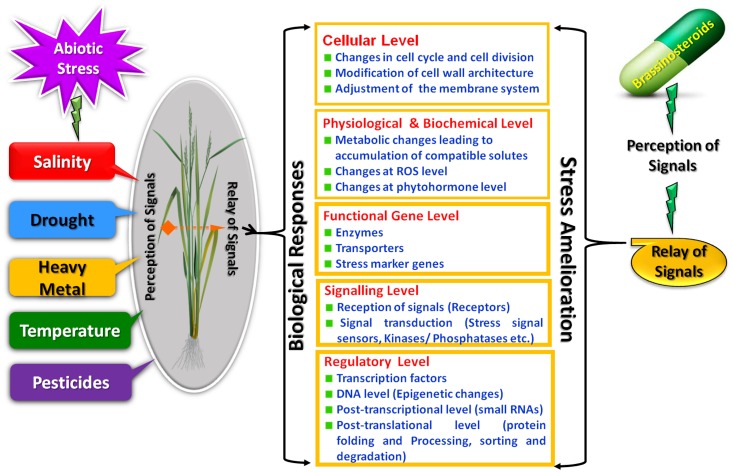
Brassinosteroids mediated remedial strategies for abiotic stress responses.

The process of a plant responding appropriately to environmental cues first requires that the plant senses the changing environment. Due to the complex nature of stress, the likely involvement of multiple sensors for perception of signals is predicted. The signal transduction pathways which are activated after the perception of signals ultimately activates stress responsive genes thus generating the initial stress response. In this update we integrate the BRs signals with various abiotic stress responses which presumably occur at different levels (**Figure 1**).

## Role of BRs at the Cellular Level

### Changes in Cell Cycle and Cell Division

The adverse effect of stress on plant growth and development could be the consequence of inhibition of the cell proliferation and cell expansion (Skirycz et al., 2011). BRs govern plant growth by regulating the process of cell expansion and division (Hacham et al., 2011; Zhiponova et al., 2013) as was clearly demonstrated by utilizing the *Arabidopsis* BR-deficient mutant, *constitutive photomorphogenesis and dwarfism* (*cpd*) (Noguchi et al., 1999). *cpd* leaf blades size was reduced in size due to decrease in cell size and number. This leaf phenotype of *cpd* was associated with prolonged cell division phase and delayed differentiation as revealed by tissue-specific marker gene expression. From this experiment, it was realized that BR production and its receptor-dependent signaling is important to differentially regulate cell division and expansion in the leaf. Since then, many reports have substantiated the role of BRs in the process of cell division and cell cycle regulation (Sun et al., 2010; Clouse, 2011; Hacham et al., 2011; Hu et al., 2017). Cell proliferation is known to be modulated by a class of protein kinases that include cyclin-dependent kinase (CDK) and cyclin which together controls the cell cycle (Kitsios and Doonan, 2011). In case of abiotic stress, the reduced expression of *CDK* genes and increased expression of their inhibitors (ICK1, EL2) to down-regulate their mitotic activity has been observed (Peres et al., 2007; Fu et al., 2008; Rodríguez et al., 2013). But investigations into the effect of BRs on cell cycle markers clearly suggests that BRs induce the expression of various cell cycle related genes (*CYCA, CYCB, CYCD3;1, CYCD3;2*) encoding cyclin dependent kinases (CDKs) and cyclophilins that play critical role in cell cycle regulation, cell proliferation and differentiation (Fu et al., 2008). Moreover, the BR activated transcription factor, BRI1-EMS-SUPPRESSOR 1 (BES1) regulates the expression of U-type cyclin CYC U4;1 and GSK-kinase to control leaf erectness by reducing the proliferation of the abaxial sclerenchyma cell number of rice lamina joints (Sun et al., 2015). Owing to the fact that rice leaf erectness improves photosynthetic efficiency, leading to enhanced yield and reduced leaf transpiration under drought stress (Lang et al., 2004), the unique role that BRs play in controlling leaf erectness makes them a key determinant for rice architecture and yield under stress conditions.

Transcript profiling and chromatin-immunoprecipitation microarray (ChIP-chip) analysis has identified a large set of genes involved in cell elongation and growth that are direct targets of the BR-regulated transcription factor BRASSINAZOLE RESISTANT 1 (BZR1) (Sun et al., 2010; Hacham et al., 2011). BRs also mediate cell cycle progression through regulation of various key transcription factors both under stressful- and normal-growth conditions. BRs regulate the R2R3-type MYB transcription factor, MULTIPASS (OsMPS), which is induced under salinity stress and negatively regulates growth by reducing cell size (Schmidt et al., 2013). Another R2R3-MYB transcription factor, BRAVO (BRASSINOSTEROIDS AT VASCULAR AND ORGANIZING CENTER) that modulates plant stem cell quiescence is regulated by BRs through the interaction with BES1. BRAVO is a cell-specific repressor of quiescent centre cell (QC) divisions in the primary root of *Arabidopsis* and counteracts BR-mediated cell division in the QC cells (Chaiwanon and Wang, 2015). BR activated transcription factor, BES1 physically interacts with BRAVO to repress its activity thus modulating the divisions of QC of the root stem cell (Vilarrasa-Blasi et al., 2014). Since the non-dividing cells in QC can initiate cell division under the effect of stress related-phytohormone and contribute to plant longevity, BRs regulation of the QC seems yet another mechanism toward providing stress acclimatization in plants (Heyman et al., 2014). As the rice QC closely resembles that of *Arabidopsis* (Ni et al., 2014), it is extrapolated that a similar mode of regulation under the effect of BRs could be expected in case of rice under stress conditions. It has been established that BRs are not only required for cell division, differentiation and expansion in the leaf, but the balance between differentiation and proliferation depends on BRI1-mediated signaling and BR levels in a temporal and spatial manner (González-García et al., 2011; Zhiponova et al., 2013). Under environmental stress, the meristematic activity in rice is maintained by a regulatory protein *RICE SALT SENSITIVE 1* (RSS1) which contributes to the vigor of meristematic cells and their viability. It regulates the G1–S transition, by interacting with protein phosphatase 1 facilitated by the phytohormone cytokinin (Ogawa et al., 2011). Though unlike cytokinin, a direct link between BRs and RSS1 has not been established, BRs alone or in crosstalk with cytokinin may be involved in regulating meristematic function in rice through the RSS1 protein and hence could be a very interesting mechanism leading to stress tolerance to explore further.

### Modification of Cell-Wall Architecture and Adjustment of Membrane System

During various stressful conditions (freezing, drought, salt stress etc.), fluctuating water or ionic status in the ambient environment poses one of the most critical and existential challenges for preservation of functionality of cellular membranes (plasma membrane and enodomembranes) of the plant cell (Golldack et al., 2014). It has been observed that BR leads to the modification of cell-wall architecture and adjustment of the membrane system thus providing a first line of defense against environmental stresses to plants (Clouse, 1996). BR signaling plays a central role in plant morphogenesis, as evidenced by various BR responsive genes linked to cell wall and the extreme developmental defects of BR mutants (Kauschmann et al., 1996). On the contrary, an enhanced BR level or activity can significantly increase plant growth, biomass accumulation, and seed yield (Salas-Fernandez et al., 2009). Global expression profiling has revealed that BRs induce the expression of several cell wall extension and loosening enzymes, e.g., xyloglucan endotransglucosylase/hydrolase (XTHs*)*, pectin lyase-like (PLLs) and expansins (EXPs) to increase cell expansion (Uozu et al., 2000; Guo et al., 2009). BES1 interacts with the rice MYB transcription factor, OsMPS to regulate the expression of expansins (OsEXPs) and endoglucanases (OsGLUs) serving as an integrative link in the crosstalk between phytohormones and the environment to regulate adaptive growth (Schmidt et al., 2013). Pectin methylesterases (PMEs) play important roles in cell wall expansion and serve as a crucial determinant of stress tolerance in rice (Yang et al., 2013). BRs signaling have been found to be indispensable for increasing PME abundance, leading to increased activity, by modulating the expression of *AtPME41* that is associated with the stress resistance mechanism (Qu et al., 2011; Wolf et al., 2012). Interference with PMEs activity (genetic or pharmacological) causes decreased pectate in the cell wall which results in stimulation of the BRI1 receptor through an unidentified feedback mechanism resulting in a compensatory up-regulation of wall remodeling agents including PMEs to protect the plant against the break-down of cell wall integrity. Recently, the primary element of this feedback loop, a RECEPTOR-LIKE PROTEIN (RLP44), has been identified (Wolf et al., 2014). RLP44 activates BR signaling through direct interaction with the regulatory BR co-receptor BAK1. Thus, RLP44 integrates the hormone signaling with cell wall surveillance to mediate wall integrity and growth. Furthermore, several genes whose products are responsible for maintaining cell wall architecture are the direct downstream targets for BR-activated transcriptional factors. One of them is the *CELLULASE SYNTHASE A* (*CESA*) family encoding a glycosyltranferase that is crucial for cellulose synthesis and is differentially regulated under various stresses (Guerriero et al., 2014). BES1 binds to the promoter regions of different sets of *CESA* genes to regulate differential responses in primary and secondary growth (Xie et al., 2011).

## Role of BRs at Physiological and Biochemical Level

Abiotic stress can confer serious damage to the photosynthesic machinery. It can damage components of the photosynthetic (PTS) apparatus directly, which leads to an imbalance in photosynthetic redox signaling resulting in the inhibition of PSII repair leading to photoinhibition (Ashraf and Harris, 2013; Gururani et al., 2015). Several studies suggest that BRs have a positive effect on the photosynthetic machinery of plants both under stress and non-stressed conditions. Exogenous application of BRs is known to alleviate photoinhibition by significantly enhancing the photochemical efficiency of PSII, the quantum efficiency of PSII photochemistry and photochemical quenching coefficient (Xia et al., 2006, 2009; Ahammed et al., 2015). BRs also significantly enhance the net photosynthetic rate, intercellular CO_2_ concentration, transpiration rate and stomatal conductance under stress (Farooq et al., 2010; Hayat et al., 2012). BRs enhance chlorophyll content and reduce the activity of chlorophyllase responsible for catabolism of chlorophyll pigment under abiotic stress (Bajguz, 2011; Hayat et al., 2012; Sharma et al., 2015). Treatment of cucumber plants with BRs up-regulated, while brassinozole (biosynthetic inhibitor of BRs), down-regulated RuBisCO and photosynthetic proteins (Xia et al., 2009). BRs induce the recovery of the photosynthetic apparatus of plants from cold stress, by eliciting the enzymes of Calvin cycle and antioxidant defense system (Jiang et al., 2013). BR application enhances ALTERNATIVE OXIDASE (AOX) activity in a RBOH-dependent manner. The enhanced AOX then contributes to balancing of chloroplast-to-mitochondria electron transfer by dissipation of excess photosynthetic reductant leading to decreased ROS accumulation and increased protection of photosystems (Deng et al., 2015).

During abiotic stress, anatomical features like stomatal and epidermal pores which facilitate gas exchange are regulated to optimize water-use efficiency and photosynthesis for stress survival. Various studies have revealed that BRs integrate with environmental signals to regulate stomatal aperture, one of the most important factors in dehydration- and salt-stress acclimatization preventing excessive water loss (Gudesblat et al., 2012; Daszkowska-Golec and Szarejko, 2013). Exogenous application of BRs promotes stomatal closure as well as inhibits stomatal opening in epidermal peels of *Vicia faba* inhibiting inwardly rectifying K^+^ channels and thus reducing guard cell uptake of K^+^ which is required for stomatal opening (Haubrick et al., 2006). Essentially, low concentration of BRs activate the RESPIRATORY BURST OXIDASE HOMOLOG (*RBOH*) genes to transiently relay hydrogen peroxide (H_2_O_2_) signal for balancing the cellular redox status of glutathione that is critical for BR-induced stomatal opening. While at higher concentration of BRs, the signal for H_2_O_2_ production is prolonged which facilitates ABA signaling and stomatal closure (Xia et al., 2014). BRs have also been known to regulate transcription factors *YODA* (YDA) and MAPK to reduce stomatal conductance and this could serve as one of the mechanism for BRs induced salt- and drought-tolerance (Kim et al., 2012). This work demonstrates that BR negatively regulates stomatal development by inhibiting BRASSINOSTEROID INSENSITIVE 2 (BIN2)-mediated phosphorylation and inactivation of YDA that leads to the activation of the MAP kinase pathway.

A common defensive mechanism against a range of stresses in rice is the accumulation of compatible solutes like proline, fructans, polyamines, myo-inositol and sugar alcohols (Kumar et al., 2013). These osmoprotectants facilitate a range of stress tolerance mechanisms involving scavenging of reactive oxygen species (ROS), maintenance of cellular turgor by adjusting osmotic balance and protection and stabilization of proteins and cellular structures. Studies show that over-expression of the biosynthetic enzyme PYRROLINE-5-CARBOXYLATE SYNTHETASE1 (P5CS1) in plants result in increased proline accumulation and enhanced stress acclimatization (Yamada et al., 2005), whereas knocking out P5CS1 resulted in impaired stress-induced proline synthesis resulting in salinity-hypersensitive plants (Szekely et al., 2008). In many studies, BRs have been shown to induce the accumulation of compatible solutes under various stress conditions which is often associated with improved stress survival (Sharma et al., 2012, 2013a; Kumar et al., 2013). Nevertheless, salt stress induced hypersensitivity of the BRs biosynthetic (*det-2*) and signaling mutants (*bin-2*) has been linked to the decreased expression of proline biosynthetic gene (*P5CS1*) and reduced proline levels which confirms the importance of BRs for inducing proline levels in stress resistance (Zeng et al., 2010). In one study, a tyrosine (Y831F) mutant of BRI1-Flag was shown to enhance the production of proline in the absence of water or osmotic stress. The authors suggest that in the absence of stress, proline may act as a signaling molecule to modify mitochondrial function and modulate gene expression resulting in an increased ability of plants to tolerate stress (Szabados and Savoure, 2010), further insinuating that the regulation of phosphorylation at the BRI1 receptor complex may be involved in enhancing abiotic stress tolerance in plants (Oh et al., 2011).

### Maintenance of Redox Potential of the Cell

Responses of plants to various types of stresses are often associated with generation of ROS, suggesting the role of ROS as a common element in plant stress signaling pathways (Liu et al., 2010; Baxter et al., 2013; Kaur et al., 2016a,b). An increasing body of evidence suggests that ROS action is bimodal: high levels of ROS cause cell death while at lower cellular levels; they play regulatory roles in plant stress responses (Baxter et al., 2013). BR-induced stress tolerance is often associated with enhanced accumulation of ROS, as ROS increased steadily with increasing concentration of exogenously applied BR (Jiang et al., 2012a,b). BRs induce H_2_O_2,_ that can behave as a signaling molecule in response to different stimuli, in turn activates MAPK that induces NADPH oxidase to self-propagate cellular H_2_O_2_ resulting in a positive amplification loop of the signal. The resultant build-up of H_2_O_2_ level up-regulates the activities of stress response related proteins, such as antioxidant enzymes, dehydrins, transcription factors, heat shock proteins induced by low-temperature and pathogenesis-related proteins to scavenge ROS, leading to suppression of ROS levels (Xia et al., 2009, 2010; Zhang et al., 2010; Cui et al., 2011; Zhu et al., 2013). Moreover, BRs cannot undergo long distance transport but they induce systemic stress tolerance by enhancing the H_2_O_2_ production (Xia et al., 2009, 2011). Inhibition of NADPH oxidase or addition of a H_2_O_2_ scavenger results in significant lowering of systemic H_2_O_2_ accumulation leading to reduced tolerance to photooxidative stress in leaves. In tomato, BRs led stress tolerance through increased apoplastic H_2_O_2_ and increased activation of MPK1/2 was hindered in *RBOH1-, MPK1/2- and MPK2-* silenced plants but not in *MPK1* silenced plants revealing a relatively more important role of MPK2 than MPK1 in BR-induced apoplastic H_2_O_2_ accumulation (Nie et al., 2013). Similarly, in *Nicotiana benthamiana*, silencing of *RBOH* compromised the BR induced AOX activity and hence reduced ROS scavanging making the plant more susceptible to abiotic stresses (Deng et al., 2015). In a recent study, BR treatment was unable to elicit antioxidant defense in the rice and maize CALCIUM/CALMODULIN-DEPENDENT PROTEIN KINASE (CCaMK) mutants. It was found that BR application results in increase in cytosolic Ca^(2+)^ concentration followed by increase in activity of CCaMK which further enhanced the BR-induced increase in cytosolic Ca^(2+)^ concentration thus forming a positive feedback loop of Ca^(2+)^ and CCaMK in BR signaling (Yan et al., 2015). ABA has also been demonstrated to be intricately involved in BR-orchestrated antioxidant defense via ZmMAP65-1a as inhibition of ABA biosynthesis reduces the expression and activity of ZmMAP65-1a (Zhu et al., 2015). The BR-induced pulse of H_2_O_2_ production via NADPH oxidase results in enhanced ABA biosynthesis, which results in further escalation of H_2_O_2_ levels leading to prolonged stress tolerance (Zhou et al., 2014). There are many reports showing BR-induced stress tolerance by regulating the activity and expression of antioxidant enzymes (Cui et al., 2015; Jin et al., 2015; Sharma et al., 2015). This is further supported by the fact that reduced BR biosynthetic mutant plants have reduced ratios of glutathione/glutathione disulfide (GSH/GSSG) and ascorbic acid/dehydroascorbic acid (AsA/DHA), while BR treatment enhances the activity of antioxidant enzymes, expression of various defense-related genes and the GSH/GSSG and AsA/DHA ratios (Sharma et al., 2012, 2013a,c; Zhou et al., 2014). Studies suggest that in rice, BR results in a concentration dependent increase in the expression and activity of antioxidant enzymes both under stress and control conditions (Sharma et al., 2012, 2013b,c; Sharma, 2014). BRs have also been shown to reduce the residues of commonly used pesticides by 30–70% in various crops including rice due to BRs induced enhanced glutathione metabolism and glutathione S-transferase (GST) activity via a *RBOH 1*-dependent pathway (Xia et al., 2006, 2009; Zhou et al., 2015).

Nitric oxide (NO) is another ubiquitous and key signaling molecule involved in various vital processes such as plant ion homeostasis, hormone responses, programmed cell death (PCD), disease resistance and responses to abiotic stress (Zhao et al., 2004). It is identified as a downstream signaling molecule of H_2_O_2_ in BR signaling and plays a critical role in BR-induced stress tolerance by modulating ROS scavenging network. Inhibition of NO production is found to inhibit BR-induced stress tolerance and partly blocks BR-induced expression and activities of various antioxidant enzymes (Cui et al., 2011; Zhang et al., 2011). Gene silencing or chemical inhibition of nitrate reductase (NR) and nitric oxide synthase (NOS)-like enzyme inhibits the BRs-induced alternative respiratory pathway and thus decreased plant’s resistance to salt stress (Zhu et al., 2015). On the other hand, BRs induce NO production in a ROS dependent manner via both NOS-like and nitrate/nitrite dependent enzymatic routes (Zhang et al., 2010; Yang et al., 2013) and NO in turn mediates induction of antioxidant genes, for mitigating oxidative stress conditions (Zhang et al., 2010). Moreover, the NO/H_2_O_2_ accumulated under BR treatment activates ABA biosynthesis which results in further enhancements of H_2_O_2_ accumulation and prolonged stress tolerance (Zhang et al., 2011). Additionally, BR induces ethylene synthesis, thereby activating GTP-binding protein Gα, that promotes *Arabidopsis* RESPIRATORY BURST OXIDASE HOMOLOG F (AtRBOHF)-dependent H_2_O_2_ production. It further elicits enhanced NO biosynthesis through NIA1 protein, augmenting signals for stomatal closure (Shi et al., 2015).

### Changes at the Phytohormone Level

Phytohormones play a central role in various plant processes involved in adaptation to environmental changes by regulating growth, nutrient allocation and source/sink interactions (Peleg and Blumwald, 2011; Tiwari et al., 2017). Abiotic stress differentially modulates phytohormonal levels by regulating their biosynthesis as well as their metabolism (Maruyama et al., 2014; Saini et al., 2015). BR signaling is known to positively regulate abiotic stress tolerance both by exogenous application of BRs (Divi et al., 2010; Sharma et al., 2013c; Wani et al., 2016) as well as by genetic deactivation of negative regulators of the BR signaling pathway (Koh et al., 2007). However, BR crosstalk with other hormones at the level of biosynthesis, signal transduction and transcriptional regulation and thus, the stress ameliorative abilities of BRs are partly derived from BR interactions with other stress related hormones (Divi et al., 2010; Choudhary et al., 2012b; Saini et al., 2013; Yusuf et al., 2017). The biosynthetic-, signaling- and constitutively active-mutants impinging on ABA, ET, JA, and SA were evaluated for heat and salinity tolerance in untreated and BR-treated samples (Divi et al., 2010). BR-conferred stress tolerance was observed to be enhanced in the ABA-deficient *aba1-1* mutant than wild type. Hypersensitivity of the *ethylene-insensitive2* (*ein2*) mutant to salt stress was rescued by BR, but BR-induced thermo-tolerance was minimal in SA genotypes *non-expressor of pathogenesis-related genes1-1* (*npr1-1*) (signaling defective for SA-mediated systemic acquired resistance (SAR). Thus, NPR1 which plays a key role in SA-mediated SAR was found to be critical to BR-mediated increase in hyper-thermal and salt-tolerance. However, BRs are also known to mitigate stress-caused oxidative damage by inducing ethylene biosynthesis (Wei et al., 2015). Upon BR stimulated induction, ethylene acts synergistically with the BRs to induce elevated H_2_O_2_ to enhance the level of AOX resulting in efficient ROS scavenging for improved stress tolerance. Similar evidence has been obtained where combined treatment of BRs with other hormones like ABA, SA, and polyamines result in a synergistic increase in stress protective effects as compared to individual hormone treatment (Choudhary et al., 2012a; Hayat et al., 2012). Microarray data also shows that BRs share overlapping regulation of many genes with other hormones like ABA, Jasmonic acid and auxin (Vert et al., 2008; Sun et al., 2010; Divi et al., 2016). However, this coordinate regulation is complex. For example, the BR interaction with ABA is multi-faceted. BR-induced accumulation of ABA content was observed in *Chlorella vulgaris* cells and in canola (*Brassica napus*) plants exposed to hyperthermal stress (Kurepin et al., 2008; Bajguz, 2009). Chemical inhibition of ABA biosynthesis led to a marked reduction in BR-induced stress tolerance (Liu et al., 2009; Zhang et al., 2011), whereas the study cited above showed ABA biosynthesis mutants had enhanced stress tolerance upon BRs application, albeit in different species (Divi et al., 2010). These reports exemplify the conflicting nature of interaction between BRs and ABA under stress. A recent study attempts to answer these discrepancies based on BRs and ABA induced H_2_O_2_ signaling under abiotic stress (Yang et al., 2014). The authors argue that continuous supply of BRs to plants result in continuous *RBOH1* induction and H_2_O_2_ production without the aid of ABA, while ABA biosynthesis becomes critical for prolonged tolerance to stress in BR-induced pathways in plants. ABA and BRs are also shown to act antagonistically on different targeted genes at or after BIN2 in BR signaling pathways and jointly fine-tune the growth of the plant when there is a competition for resources between stress responses and growth (Chung et al., 2014). Thus, under natural conditions, different hormonal signaling pathways undergo a crosstalk among themselves and among various stress signaling pathways in combinations to generate a customized stress response that is most suitable for the survival of plant under given environmental conditions.

Genetic and physiological studies have shown that BRs and auxin interact antagonistically in roots to maintain the spatiotemporal balance between stem cell division and differentiation required for optimal root growth (Chaiwanon and Wang, 2015). Interestingly, BRs have a dual effect of both delay and promotion of stem cell differentiation, depending on the site of their action. BR-induced genes mostly occur in epidermal cells of the basal meristem zone which also happen to be enriched with auxin related genes. While BR down-regulated genes occur in the stele of the apical meristem zone and thus regulate differential growth (Vragović et al., 2015). Similar integration of BRs signaling pathway with that of other phytohormones explains differential spatiotemporal context of brassinosteroid activity in a tissue and organ specific manner (Singh and Savaldi-Goldstein, 2015).

BRs and GA work antagonistically to provide submergence tolerance in lowland rice plants (Schmitz et al., 2013). While floating- and deepwater-rice cultivars avoid submergence by exaggerated stem elongation, lowland rice cultivars have been demonstrated to have enhanced survival of flash-flooding episodes by repressing stem elongation. Repression of stem elongation is thought to provide reserves for maintenance of cellular homeostasis during the period of low gas exchange (Louis Setter and Vidonia Laureles, 1996). Exogenous BRs induce ancillary stabilization of the DELLA proteins and SLENDER RICE1 (OsSLR1*)*, a GA signaling repressor resulting in reduced shoot elongation correlated with improved submergence tolerance. BRs also crosstalk with cytokinin to induce drought stress tolerance (Peleg et al., 2011). Enhanced expression of ISOPENTENYL TRANSFERASE, a key enzyme in cytokinin synthesis, the gene of which, when under the control of a drought inducible- or senescence inducible-promoter, resulted in increased tolerance to drought with concomitant increases in genes encoding proteins involved in both BR biosynthesis and signaling, suggesting a cross-talk between BRs and cytokinin leading to drought stress tolerance. A large number of BR regulated genes are also regulated by polyamines (spermidine) and simultaneous application of both phytohormones generate additive or synergistic effect to enhance heavy metal tolerance of radish plants relative to the application of each hormone individually. The resulting tolerance was partly attributed to regulation of genes involved in auxin and ABA metabolism under the combined application of BR and spermidine, which could be contributing toward improved seedling growth and normal functioning of several physiological processes, such as seed germination, regulation of stomata and embryogenesis(Choudhary et al., 2012a).

## Role of BRs at Functional Gene Level

Various genes have been identified in rice to be induced under abiotic stress. Functional annotation of these genes reveal that they are involved in various cellular processes, such as vesicle trafficking, signaling, cytoskeleton rearrangement, and biosynthesis of hormones and vitamins (Basu and Roychoudhury, 2014; Qian et al., 2015; Yang et al., 2015). Many of these gene products are also regulated by BR viz., molecular chaperones (heat shock proteins), cytoskeleton proteins (tubulin, actin), redox metabolism (dehydrins, glutathione-S-transferase) and hormonal biosynthesis and metabolism (ACC oxidase and allene-oxidase cyclase) (Deng et al., 2007; Sun et al., 2010; Divi et al., 2016) which play important roles in plant growth and development (**Table 1**). BR transcriptome analysis has identified abiotic stress-related alteration in transcription of *JACALIN-RELATED LECTIN1-3* (*JAC-LEC1-3*), *WRKY33*, *ACID PHOSPHATASE5 (ACP5)* and a *BR-RESPONSIVE-RECEPTOR-LIKE KINASE* (*BRRLK*) (Divi et al., 2016). Genetic and chemical evidences have identified various important genes like *DWARF AND LOW-TILLERING (DLT), LEAF AND TILLER ANGLE INCREASED CONTROLLER (LIC), DWARF1 (D1)* and *TAIHU DWARF1 (TUD1), CYP90D2/D2* which play crucial role in maintaining several features of rice architecture are also regulated by BRs during stress (Wang and Li, 2005; Li et al., 2013; Zhang et al., 2014). Recently, a rice microRNA, *OsmiR397*, has been shown to positively control rice grain size and yield by down-regulating its target, *OsLAC*, which encodes a laccase-like protein that regulates BR sensitivity (Zhang et al., 2013). Another protein, TUD1, encodes a functional U-box E3 ubiquitin ligase has been reported to be an interactor of proteins OsD1/RGA1, which forms the alternate pathway for BR signaling in rice. In rice, gene products like heat shock proteins (OsHSP17.0 and OsHSP23.7) that are implicated in the proper folding, stabilization, maintenance and degradation of numerous proteins, are known to be up-regulated under abiotic stress conditions and have recently been found to be direct targets of BZR1/BES1 (Kagale et al., 2007; Shigeta et al., 2014). Amino-terminal and central parts of BES1 provide affinity for physical interaction with HSP90.3 *in vitro*. In addition to BES1 and BIN2 being binding partners of HSP90, BZR1 has been identified as a novel HSP90 target and thus signifies that HSP90 forms a complex with two major transcription factors in BR-mediated gene expression (Shigeta et al., 2015). Rice plants accumulate LATE EMBRYOGENESIS PROTEINS (LEA), which provide stress protection by preventing protein aggregation and resulting in stabilization of membranes, are also targeted by BR-signaling activated BES1 (Li et al., 2009; Duan and Cai, 2012). The transcript level of *RESPONSIVE TO DESICCATION 29A* (*RD29A*) and *EARLY RESPONSE TO DEHYDRATION10* (*ERD10*) encoding LATE EMBRYOGENESIS PROTEINS are enhanced upon BR treatment (Kagale et al., 2007). Proteomic approaches have also identified several proteins which are important for BR-induced stress tolerance (Deng et al., 2007; Sharma et al., 2013c). ABSCISIC ACID STRESS RIPENING (ASR)-like protein and LIPOCALINS which play essential roles in signal transduction under cold stress; REMORIN, an important membrane skeleton protein which is associated with biotic- and abiotic-stress responses as well as drought-stress responsive-and dehydrin-proteins are some of the key proteins involved in BRs induced stress tolerance (Li B. et al., 2012). The protein level of FERRITIN, a ubiquitous multimeric iron storage protein which helps sequester excess free iron is induced under the effect of salt or brassinolide treatment in *Arabidopsis* (Sharma, 2014). It might be a mechanism not only for maintaining iron homeostasis but for prevention of the formation of hydroxyl radicals through the Fenton action as well and thus it might be helping maintain ion balance and general enzyme activity (Lobréaux et al., 1995) under stress conditions. An interesting study was performed on the *SALT* gene which is located on the *SalTol* QTL in rice and expressed highly under the effect of salinity (Sharma et al.,2013c; Sharma, 2014). While the expression of the *SALT* gene rose under salt treatment, its expression was severely reduced in rice seedlings co-treated with salt and BRs. The promoter region of the *SALT* gene revealed the presence of motifs such as ABRE (abscisic acid responsive element), MBS (a MYB binding site involved in drought-inducibility), LTR (involved in low-temperature responsiveness), that are implicated in different kind of stresses. Thus, the presence of these motifs in the *SALT* promoter and *SALT’S* regulation by BRs can be explored as one of the mechanisms by which BRs induced stress protection to a multitude of extrinsic factors (Sharma, 2014). Furthermore, manipulation of BR biosynthetic genes has also provided evidence for the role of endogenous BRs in stress tolerance. Application of brassinozole, the specific inhibitor of the DWARF4 (DWF4), a protein involved in BR-biosynthesis results in plants being susceptible to stress (Xia et al., 2009), while overexpression of the BR-biosynthetic genes (*AtDWF4* and *HYDROXYSTEROID DEHYDROGENASE1)* in *Arabidopsis* seedlings result in enhanced tolerance to abiotic stress relative to wild-type seedlings (Divi et al., 2010; Li et al., 2007). Ecotopic expression of AtDWF4 in *Brassica napus* revealed that BR mediated growth enhancement and defense responses are likely to be mediated by BES1/BZR1. Further, it was established that BR can simultaneously regulate abiotic stress tolerance and plant productivity (Sahni et al., 2016). In a contradictory study, RNA interference–mediated interruption of the rice SQUALENE SYNTHASE (SQS) catalyzing the initial reaction in the isoprenoid metabolic pathway for sterol (including brassinosteroid) synthesis resulted in reduced stomatal conductance and improved drought tolerance (Manavalan et al., 2012) suggesting that reduced sterol and BR levels might enhance stress tolerance leading to a skepticism in acknowledging the role of endogenous BRs in stress tolerance (Jager et al., 2008). Furthermore, rice BR biosynthetic mutant *d2-1*, exhibited enhanced tolerance to Fe-deficiency and this response could be reversed partially by exogenous BR application. Authors argue that the lower endogenous BRs facilitates Fe transport and translocation from roots to shoots while complementing the seedlings with BR suppresses the translocation of Fe from roots to shoots, displaying a severe phenotype of Fe deficiency in shoots (Wang et al., 2015). Nevertheless, the stress response may not be manifested in the form of changes in BR levels but could be reflected in changes in the activity (sensitivity) of BR signaling components as explained by Xia et al. (2009). There are several other important stress regulated genes like *CHLORIDE CHANNEL* (*CLC*), transporters and symporters (OsAKT, OsHKT, OsNHX), *NON-SELECTIVE CATION CHANNEL* (*NCC*), *MYO-INOSITOL SYNTHESIS* (*PINO1*), etc. which play pivotal roles in stress tolerance (Kumar et al., 2013). Further characterization of these genes under the effect of BRs will improve our understanding of their role in BR-mediated stress tolerance.

**Table 1:** Major stress responsive genes regulated by Brassinosteroids.

Gene	Function	Stress type	Reference
*DREB*	Transcription factor that regulates different cold stress responsive genes	Drought stress	Kagale et al., 2007; Xiao et al., 2009
RESPIRATORY BURST OXIDASE HOMOLOG *( RBOH )*	ROS generation	Photo-oxidative and cold stresses	Xia et al., 2009
*PYRROLINE-5-CARBOXYLATE SYNTHETASE1 (P5CS1)*	Proline biosynthesis	Salinity stress	Zeng et al., 2010
*DELLA*	Gibberellic acid signaling repressor	Submergence stress	Peleg et al., 2011
*SLR1*	Gibberellic acid signaling repressor	Submergence stress	Peleg et al., 2011
*YODA* (YDA)	Transcription factor that regulate stomatal conductance	Salinity and drought stress	Kim et al., 2012
*ABSCISIC ACID STRESS RIPENING (ASR)*	Signal transduction under cold stress	Cold stress	Li B. et al., 2012
*LIPOCALINS*	Signal transduction under cold stress	Cold stress	Li B. et al., 2012
*REMORIN*	Membrane skeleton protein	Drought stress	Li B. et al., 2012
*MULTIPASS (MPS)*	Transcription factor that regulates cell size	Salinity stress	Schmidt et al., 2013
*ALTERNATIVE OXIDASE (AOX)*	Protection of plant photosystems	Cold stress	Jiang et al., 2013; Deng et al., 2015
*FERRITIN*	Iron storage	Salinity	Sharma, 2014
*SUPEROXIDE DISMUTASE (SOD)*	H_2_O_2_ biosynthesis	Salinity and Pesticide stress	Sharma et al., 2012, 2015
*ASCORBATE PEROXIDASE (APX)*	ROS scavenging	Salinity and Pesticide Stress	Sharma et al., 2012, 2015
*CATALASE (CAT)*	ROS scavenging	Salinity and Pesticide Stress	Sharma et al., 2012, 2015
*GLUTATHIONE REDUCTASE (GR)*	ROS scavenging	Salinity and Pesticide stress	Sharma et al., 2012, 2015
*NON-EXPRESSOR OF PATHOGENESIS-RELATED GENES1-1* (*NPR1-1*)	Transcription factor that regulates different stress responsive genes	Hyper-thermal and Salinity stress	Wei et al., 2015
*WRKY*	Transcription factor that regulates different stress responsive genes	Drought stress	Chen et al., 2017
*CESTA (CES)*	Transcription factor that regulates different cold stress responsive genes	Cold stress	Eremina et al., 2017

### Role of BRs at Signaling Level

Research performed in the domain of BR induced stress response and tolerance in the past two decades has provided overwhelming evidence to support the involvement of various key players of BRs signaling in abiotic stress amelioration. One of the remarkable studies conducted by Cui et al. (2012) provide evidence that BR signaling through BRI1 and its quality control is necessary for salt stress tolerance in *Arabidopsis.* Mutant BRI1 (*bri1-9* and *bri1-5*) are retained and degraded by the endoplasmic reticulum (ER)-associated protein degradation (ERAD) pathway system. Mutation of *UBC32*, a functional component of ERAD, is involved in positive regulation of BR signaling pathway through the buildup of the BRI1-9 protein on the plasma membrane. The salt tolerance test conducted on *bri1-9* and *bri1-9 ubc32* double mutant plants revealed that *bri1-9* plants were more salt-sensitive relative to wild-type plants while *bri1-9 ubc32* double mutants partly rescued the salt-sensitivity of *bri1-9*. Further, the *Arabidopsis* BR-insensitive mutant *bin2-1* was found to be hypersensitive to salinity stress which was correlated with inhibited induction of stress responsive genes (Zeng et al., 2010). Similar scenarios were observed in rice and *Arabidopsis* where the combined application of exogenous BRs and salt treatment results in significant up-regulation of transcript and protein level of OsBRI1 and BRI1 as compared to individual treatments of salt or BR, hinting toward a synergistic regulation of the BR receptor at the gene and protein level by salt and exogenously applied BRs (Sharma et al., 2013c; Sharma, 2014). Though these results were in contradiction to the previous studies which show down-regulation in OsBRI1 expression under BR treatment (Yamamuro et al., 2000; Tanabe et al., 2005). This could be due to the difference in the mode of treatment of BRs. In our work (Sharma et al., 2013b) a short-term (8 h) soaking treatment of rice seeds in 10^-7^ M EBL was provided while in the other reports seeds were grown on agar medium containing 10^-6^ M BL for a period of 2 weeks or 10 days suggesting that BR signaling might be differentially modulated by prolonged and short-term exposure to BRs. It has also been shown that prolonged elevated temperature conditions led to increase in root growth mostly independent of auxin, by ubiquitination mediated degradation of BRI1 to down-regulate BRs signaling (Martins et al., 2017). While homologous mutant alleles of BRI1 (*bri1-9* and *brl3*) manifest an increased tolerance to cold stress (Kim et al., 2010), constitutive activation of BR signaling pathways due to gain-of-function of an allele of BAK1 in *Arabidopsis*, *elongated-D (elg-D*) resulted in plants with reduced ability to resist stressful conditions though they displayed improved growth (Chung et al., 2012). Therefore, it is likely that plants might be undergoing a trade-off between growth and stress responses as BR-mediated growth promotion might be occurring due to re-allocation of energy that is normally utilized in defense related pathways. A recent study has shown that BR and autophagy pathway are integrated among each other to coordinate growth and stress response. BIN2 phosphorylates ubiquitin receptor protein DSK2, which targets BES1 for degradation and results in reduced plant growth. On the other hand, accumulation of BES1 in loss-of-function *dsk2* mutants result in compromised drought and starvation responses (Nolan et al., 2017). Another bridge between BRs signaling and stress acclimation is the process of Regulated Intra-membrane Proteolysis (RIP). RIP controlled by ER stress is a conserved response mechanism in eukaryotes. Various stresses (heat, salinity, inhibition of protein glycosylation) lead to an enhanced translocation of transcription factors (bZIP17 and bZIP28) from the ER through the golgi into the nucleus which activates BR signaling besides activating ER chaperone genes. Under abiotic stress, RIP–dependent ER chaperone synthesis, may promote correct folding or modification of BRI1 which might have undergone impaired maturation and translocation due to stressful conditions. Thus, RIP may enhance BR signaling directly, and promote stress tolerance by enhancing BRI1 localization to the plasma membrane (Che et al., 2010).

In addition to the BR receptors, BR Signaling Kinases (BSKs) are the key components of BR signaling, involved in development and stress signaling in plants. Their BR signaling function is conserved in *Arabidopsis* and rice. OsBSK3 has been found to be a positive regulator of BR signaling in rice and directly interacts with OsBRI1, undergoes phosphorylation and transduces the signal to downstream signaling component (Zhang et al., 2016). The transcript level of BSK5 was induced by stimuli like salt, drought, BRs and ABA while the loss of function mutant *bsk5* exhibited sensitivity to salinity and ABA. Since, BSKs are known to be positive regulators of BRs signaling, the resulting sensitivity of the *bsk5* mutant to stress may be due to repression of BSK5 leading to BR-deficiency that affects salt- and ABA-stress responses (Li Z.Y. et al., 2012). OsGSK1, a GSK3/SHAGGY-like kinase is a homologue of a key component of BR signaling in *Arabidopsis*, BIN2 (a negative regulator of BRs signaling). OsGSK1 knock-out plants were found to be hypersensitive to BRs and had significantly enhanced tolerance to various abiotic stresses with altered expression of various abiotic stress responsive genes (Koh et al., 2007).

## Role Of Brs At Regulatory Level

**FIGURE 2:**
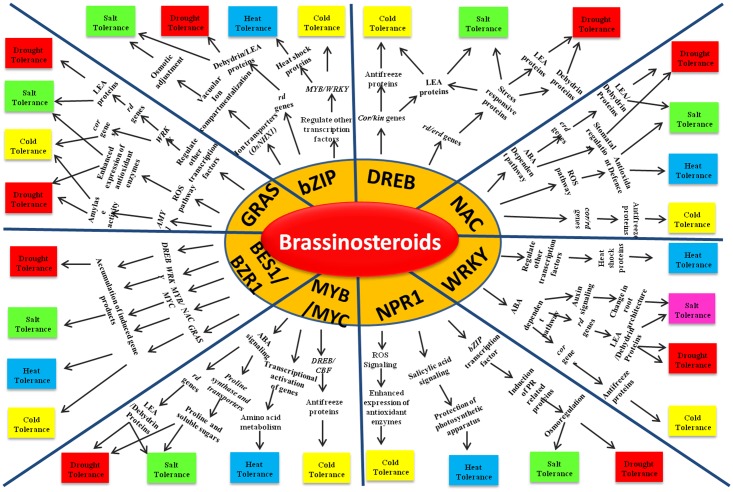
Abiotic stress tolerance in rice by interaction between brassinosteroids and various transcription factors.

Brassinosteroids directly or indirectly regulate different stress responsive transcription factors through their signaling module operated through negative regulator BIN2 and key transcription factors (TFs) BZR1/BES1, leading to the activation of stress adaptive signaling pathways (**Figure 2**). DREB, WRKY, MYB/MYC, GRAS, bZIP, NAC and NPR are the major transcription factors regulating abiotic stress responses in rice. Over expression of DREB in transgenic rice has been found to confer tolerance to salt, drought and cold stress by activating a cascade of genes including RD, ERD and KIN (Chen et al., 2008; Wang et al., 2008). BRs have been known to enhance the expression level of DREB under the normal as well as stressed conditions leading to stress adaptation (Kagale et al., 2007; Xiao et al., 2009). WRKY is another critical component of the complex stress responsive signaling pathways in rice and its expression is found to be induced by BR treatment in plants (Xiao et al., 2009; Chen et al., 2012). *OsWRKY08* confers salt, cold and drought tolerance by regulating auxin signaling and stress responsive genes, e.g., *RD* and *COR* (Yu et al., 2010; Chen et al., 2012). The *Os*MYBS3 TF is reported to confer chilling tolerance in rice by regulating DREB/CBF TFs and the cell cycle (Ambawat et al., 2013). MYB/MYC TFs also regulates amino acid metabolism and confers heat tolerance in rice (El-kereamy et al., 2012). Microarray and genome wide expression studies have indicated that expression of this class of TF is regulated by BRs (Goda et al., 2002; Xiao et al., 2009). Recently it is evidenced that the BR regulated transcription factor CESTA (CES) contributes to the constitutive expression of the transcription factor CBF that control cold responsive (COR) gene expression. Furthermore, *COR* genes that are regulated in a CES- and BR- dependent manner but independent to CBF regulation have also been identified during cold acclimation. It is predicted that BRs induce posttranslational modification of CES and its other redundant TFs which further regulates cold-responsive TFs to induce basal resistance against freezing stress leading to cold acclimation (Eremina et al., 2017). The GRAS domain containing TFs are plant specific playing an array of roles in development and positively regulating the salt, cold and drought stress tolerance in plants (Xu et al., 2015). GRAS TFs are reported to be involved in the BRs signaling pathway (Tong et al., 2009). Over expression of the NAC TFs can improve the salt, cold, drought and heat tolerance of rice (Jeong et al., 2010; Hong et al., 2016). They confer heat and drought stress adaptation by increasing the activity of ROS detoxification enzymes in rice and cold tolerance through the regulation of *COR* genes (Fang et al., 2015). Brassinosteroids may regulate NAC TFs during abiotic stress tolerance in crosstalk with other plant growth regulators like ABA (Fujita et al., 2004; Ahammed et al., 2015). RD26 which belongs to the family of NAC TFs, regulates drought-responsive gene expression. A recent study has shown that BES1/BZR1 directly targets and represses the expression of *RD26* at a transcription level, while RD26 interacts with BES1 to inhibit its transcriptional activity. Such a mutual inhibitory mechanism not only result in inhibition of BR-induced growth under drought conditions but also prevents the unwanted elicitation of drought response during BR-induced growth (Ye et al., 2017). Also, a NAC TF, JUB1 has been found to regulate GB/BR signaling and repress TF PIF4 thereby connecting hormonal and environmental stimuli (Shahnejat-Bushehri et al., 2016). Although, NPR1 is predominantly known for its role in biotic stress response, its role in abiotic stress adaptation is also well explored. NPR1 expression is positively regulated by brassinosteroids that confers abiotic stress tolerance in a salicylic acid mediated pathway (Saini et al., 2015). Overexpression of *AtNPR1* gene in rice results in the enhanced expression level of PR proteins and antioxidant enzymes that protects the plants against oxidative stress (Srinivasan et al., 2009*).* NPR1 also interacts with the TF bZIP and induces the expression of PR proteins which confers salt and drought tolerance in rice through osmoregulation (Akiyama and Pillai, 2001; Srinivasan et al., 2009; Alves et al., 2013). bZIP TFs are one of the largest families of plant TFs and have been found to be indirectly regulated through brassinosteroids in a NPR1 mediated pathway for conferring abiotic stress tolerance in *Arabidopsis* (Divi et al., 2010; Liu et al., 2014). Overexpression studies with *OsbZIP71* has resulted in the enhanced salt- and drought-tolerance in rice through the increased expression of stress responsive genes like *NHX1* which is involved in ion compartmentalization and *RD* genes encoding dehydrins. Its overexpression further enhanced the expression level of *OsMYB4* that encodes for a stress responsive TF and regulates abiotic stress responses in rice (Liu T.T. et al., 2013) and are also known to be regulated by BR signaling (Schmidt et al., 2013). As BRs regulate different TFs to confer abiotic stress tolerance in rice, it will be interesting to further explore the pathways to determine how BES1/BZR1 relays signals for the activation of these TFs

Epigenetic regulation has been known to be associated with the expression changes in stress-responsive genes (Luo et al., 2012; Kim et al., 2015). BR regulated BES1 interacts with a pair of histone demethylases and also recruits histone lysine (Wang et al., 2014) suggesting that epigenetic modifications might also regulate the expression of a subset of BR targeted genes. In rice, H3K36 methyltransferase encoded by SDG725 is important for the methylation of histone lysine residues. Downregulation of SDG725 resulted in various growth defects which were reminiscent of BRs knock-down phenotypes. Transcripts of many BR signaling and synthesis genes (*OsDWARF11*, *BRI1* and *BU1*) were reduced more than two-fold upon depletion of SDG725. Furthermore, a depletion of H3K36me2/3 in chromatin of BR-related genes was observed in *sdg725* knock-down plants. Moreover, the SDG725 protein binds directly to BR regulated genes revealing that SDG725-mediated H3K36 methylation is indispensable for the expression of BR regulated genes to modulate plant growth and development (Sui et al., 2012). Further, genome-wide transcriptome analysis in plants reveals that under abiotic stress conditions large amount of non-coding RNAs (ncRNAs), small nuclear RNAs (snRNAs) and small nucleolar RNAs (snoRNAs) are expressed (Liu T.T. et al., 2013) and possibly they have a role in chromatin regulation, translational repression and regulation of RNA stability during stress response (Di et al., 2014). Hence, it is equally interesting to look at the modulation of these snRNA by BRs and their implication in abiotic stress tolerance.

Recently there has been a huge interest in studying abiotic stress in the context of protein folding, stabilization and dynamics (Ortbauer, 2013). The association of BR signaling component (BES1) with Heat shock protein 90 (HSP90) which is a known molecular chaperon and implicated in protein folding, stability, trafficking and degradation provides evidence for an indirect link of possible involvement of BRs mediated protein folding and abiotic stress tolerance (Shigeta et al., 2014). Besides protein conformation, BRs also regulate the other post-translational modifications of proteins like phosphorylation, ubiquitination and sumoylation which play critical role in abiotic stress tolerance in plants (Kelley and Estelle, 2012; Toit, 2014). Furthermore, the regulation of BR receptors is also believed to be critical for the response of the plant to various stresses. Two important processes that regulate the functioning of BR receptor are clatharin mediated endocytosis (CME) and phosphorylation of the kinase residues on BRI1 (Clouse, 2011; Martins et al., 2015). CME in association with a network of proteins, including clathrin, adaptors, and accessory proteins are involved in selection and recruitment of cargos which are further fated for recycling or degradation (Di Rubbo et al., 2013). Salinity induced endocytosis has been implicated in adaptation of plants to stresses (Galvan-Ampudia et al., 2013). Though various genetic, biochemical and advanced microscopy studies have led to the establishment of endocytosis as a crucial process that regulates many diverse aspects of plant life but, a knowledge gap exists in integrating CME of BRI1 in response to diverse environmental cues and their implications in stress adaptation. Furthermore, to the best of our knowledge, no study has been conducted so far to examine the changes in the phosphorylation status of the BRI1 receptor complex under the effect of stress. As BAK1 has been found to bind to FLAGELLIN-SENSITIVE 2 (FLS-2) to regulate biotic stress responses (Chinchilla et al., 2007; Lozano-Durán and Belkhadir, 2017), it is quite plausible that BRI1 could also be acting as a co-receptor to specific molecules of salt or some xenobiotics to modulate BR signaling pathway. Such studies could help immensely in deciphering novel mechanisms of BR related stress responses.

## Future Prospects and Conclusion

**FIGURE 3:**
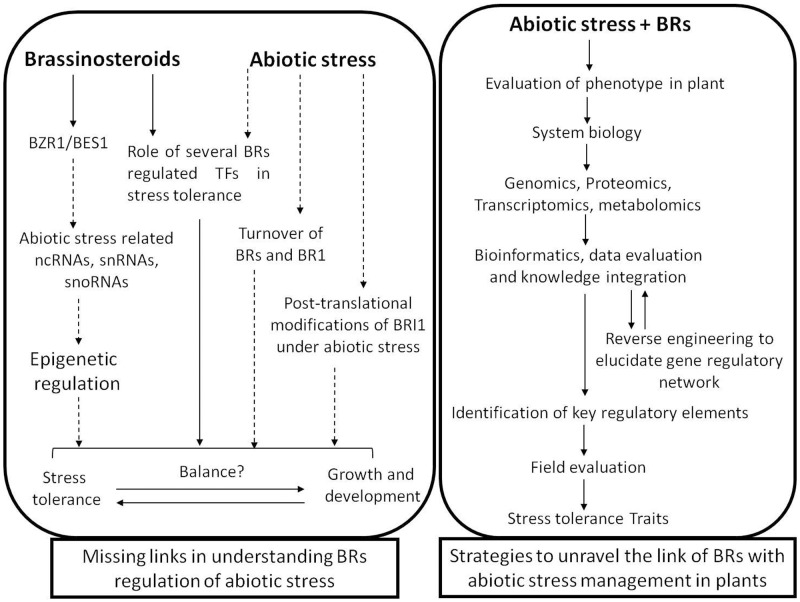
A schematic representation of the missing links in understanding brassinosteroids regulated stress tolerance mechanism and the strategies to address them. Solid lines represent the well-established concepts and dashed lines represent the open questions.

Conferring abiotic stress tolerance in plants is a fairly complex process, and diverse mechanisms are being explored which are linked to this event. At present, there are many reports establishing the role of BRs in modulating stress related responses in plants. However, the intricate mechanisms associated with such responses in plants are still elusive (**Figure 3**). In recent years, there is substantial progress in understanding various components associated with the perception of BR signals and their transduction, yet the interlinking of these signals leading to abiotic stress tolerance in plant is still a subject of great interest. Deeper insights into the actions of BRs at multiple levels and the integration of these inputs will probably provide a road-map for addressing the problem more holistically. Further, the dynamics of BR homeostasis which is greatly dependent on its synthesis, degradation and transport need to be explored in the context of abiotic stress and other environmental stimuli. It is also of great relevance to understand the interplay of various phytohormones with BR which ultimately broadens its biological functions. The insights into such crosstalk at signaling level needs to be investigated and understanding their modulation by abiotic stress will lead to addition of knowledge in the area. Moreover, with rapid development of genomics and proteomics technologies leading to identification of key genes and proteins related to stress responses in plants provide a suitable platform to explore the role of BR signaling in stress amelioration. Furthermore, addition of knowledge on transcriptional and post-transcriptional as well as translational and post-translational events regulated by BRs will be critical in modulating the regulatory role of BRs in abiotic stress tolerance in plants.

## Author Contributions

All authors listed have made a substantial, direct and intellectual contribution to the work, and approved it for publication.

## Conflict of Interest Statement

The authors declare that the research was conducted in the absence of any commercial or financial relationships that could be construed as a potential conflict of interest.
